# The importance of having a paid job. Gendered experiences of health and ill-health in daily life among middle-aged women and men

**DOI:** 10.1186/s12889-021-12034-7

**Published:** 2021-11-06

**Authors:** Anne Hammarström, Berit Lundman, Astrid Norberg

**Affiliations:** 1 IMM, Karolinska Institutet, Stockholm, Sweden; 2 Department of Epidemiology and Global Health, Umeå University, Umeå, Sweden; 3 Department of Nursing, Umeå University, Umeå, Sweden

**Keywords:** Paid work, Domestic work, Social determinants of health, Gender relations

## Abstract

**Background:**

More gender-theoretical studies are needed to gain a deeper understanding of what life circumstances make people sick or improve their health. The aim of the study was to gain a deeper understanding of social determinants of health by exploring gendered experiences in daily life among middle-aged women and men using the theory of gender relations.

**Methods:**

Individual interviews with nine men and women were performed, focusing on what made them feel good or bad. Qualitative content analysis was used to analyse the data.

**Results:**

A major theme in our interviews was the gendered health-promoting experiences related to having a job, which involved becoming someone, feeling appreciated at work and having control over work. Having good family relations was also health-promoting, in terms of supportive relations and becoming a parent. Ill-health was related to gendered adverse conditions at work (accidents, monotonous and stressful work tasks, being bullied) and in domestic life (demands, destructive partner relations, having children with problems).

**Conclusions:**

Gendered determinants of health and ill-health were identified in both working and domestic life. Public health policy needs to challenge the gender order in society, which defines the gendered structure of the labour market as well as the gendered relations in domestic life.

## Background

Research in public health and medicine is often descriptive and oriented towards an individual level [1, 2]. Theories are needed to gain a deeper understanding of what life circumstances make people sick or improve their health. But there is a lack of theories in public health and in much of the existing medical research [1, 2]. As life circumstances often are gendered (i.e. differ in distribution between men and women) there is a need for gender theories in order to understand what makes men and women sick [3].

All societies have cultural accounts of gender, from which various forms of masculinities and femininities are constructed [4]. Gender is a relational concept, where masculinities are constructed in contrast to femininities [4]. A useful theoretical framework for public health research on men’s and women’s health is the relational theory of order, developed by Raewyn Connell [5]. Here, gender is understood as the relations – in any given society – between men and women, with the focus on economic relations, power relations, affective relations, and symbolic relations [5]. The focus here is on the uneven and gendered distributed life circumstances that result in women’s poorer work environment and higher/total responsibility for unpaid domestic work.

The WHO report on Social Determinants of Health [6] concluded that the health of a population depends on the living conditions in which people grow, live, work and age, and the systems that deal with illness. Thus, alongside childhood conditions, there is a need to emphasise housing, domestic and waged work, employment conditions, lack of power and resources. Gender was absent from the report except in one chapter from the ‘Women and Gender Equity’ network. However, that chapter has been heavily criticised by researchers for not making use of gender theories [3, 5]. The report has also been criticised for ignoring the fact that social determinants are shaped by other power relations such as social class [7].

The report from the WHO commission calls for a better understanding of social determinants of health. Qualitative designs and theories are needed to arrive at a deeper understanding of social determinants, beyond simple predictive factors or correlations [8]. But still today there are few qualitative studies in the field. A cross-sectional qualitative study among adolescents from Indonesia showed that family connections (particularly with parents), school pressures, and adverse exposure on social media were important drivers of poor mental health [9]. Another study with the same design among Chinese older adults identified the following social determinants of depressive symptoms: societal conflicts, family conflicts, financial constraints, personality and worsening physical health [10]. Both these empirical studies applies social frameworks but neither of them used any theories. Other qualitative studies dealt with specific diseases (such as disabilities, diabetes, HIV) or focused on interventions and policymakers. Thus, there is a need for theoretically driven qualitative studies about social determinants of health.

We have identified only a few qualitative studies about social determinants of health, based on gender theories. One [of them, based on feminist theory, analysed interviews among 20 elderly Swedish women and found that paid work was experienced as health-promoting through giving meaning, self-esteem, social relations, a room of one’s own, a basis for self-determination and independence (from men), freedom from constant availability for the needs of their families, and it strengthened them in unequal, or even abusive, partner relationships [11]. Another theoretically driven paper showed that stereotypical gender practices in housework can increase experiences of stress among women and men. Challenging stereotypical masculinities can be crucial for breaking the process of resignation in housework and for facilitating improved health among both women and men in heterosexual couple relationships in a Swedish context [12]. A qualitative study of Canadian men explored the experiences of gendered work-place bullying, using theories based on constructions of masculinities [13]. The main problem experienced was a lack of workplace support to address and prevent the bullying. The health consequences of such bullying were identified and contrary to common beliefs, the men sought help to address the problems. Another Canadian qualitative study, integrating gender theories such as doing gender and gender constructions throughout the paper, identified how gyms become places where gendered inequalities in health emerge [14]. The study identifies how micro-level gendered processes at the gym constructs gender disparities in physical activity between men and women.

More gender-theory-based studies are needed. Therefore, the aim of the study was to achieve a deeper understanding of social determinants of health by exploring gendered experiences in daily life among middle-aged women and men using the theory of gender relations.

## Method

A qualitative method was chosen to capture the complex process by which individuals relate experiences in domestic life and in working life to their own well-being and health.

### Participants

Participants were selected from the Northern Swedish Cohort (NoSCo), which consists of all pupils (*n* = 1083, age 16) in the last year of compulsory school in a middle-sized town in Northern Sweden. Like Sweden as a whole, the labour market in the area was gender-segregated, with a men-dominated workforce at employers such as a steel company, a technical university college and the Swedish Armed Forces, and women-dominated employees in health care, elderly care, schools, and preschools.

The cohort has been followed with comprehensive questionnaires at regular intervals in 1983, 1986, 1995 and 2008. The questionnaires were developed from previously validated questions on topics such as socio-economic conditions, self-rated health and health behaviour [15]. The participation rate in the cohort has been extremely high during all follow-ups, and after 27 years 94.3% (*n* = 1010) were still actively participating.

For this qualitative study, a subsample was selected from the cohort in purposeful way. The PI of the cohort had the possibility to use the quantitative questionnaire data from age 16 and age 43, to identify participants with the highest increase respectively decrease of symptoms of mental health during this 27-year period. Among those with the highest increase respectively decrease she made a strategic selection of men and women with various degree of education.

In that way, four men and five women with various educational background in combination with major variations in symptoms of mental health were selected. Three participants refused to take part in the interviews. Of the women three had longer education and two shorter and of the men two had longer and two had shorter education.

Thus, the sample of this qualitative study consists of 5 women and 4 men, who were 50 years old.

### Data collection

The participants were informed that the aim of the interview was to increase our understanding of what is of most importance for their health status during different periods of life.

Experienced interviewers performed the interviews. One informant was interviewed twice to support her and to follow up her mental health problems. Neutral places such as libraries and conference rooms were chosen for the interviews. The interviews lasted for one to three hours each and were audio-recorded. To facilitate recollection of occurrences/events and their sequencing, a lifeline was used. The interviewer gave the participant a pencil and a piece of paper and asked them to illustrate the development of their health during life. The interviewer described how the lifeline should be constructed, with their age on the x-axis and perceived health on the y-axis (good health at the top, health as usual in the middle and bad health at the bottom). If necessary, the interviewer drew the x- and y axes. The participants were asked to draw how their health had developed from the first age they could remember until the day of the interview. The informants were asked to use the lifeline to reflect on their life and narrate “Specific occurrences in their life in relation to each period of improved respectively deteriorated health”, “Why did their health improve or deteriorate at this specific time of their life? At periods of deteriorated health, they were also asked to narrate: “Strategies used to overcome difficulties”.

### Data analysis

Data analysis followed the steps of the analytical process described for qualitative content analysis [16]. The method is used in systematic analysis of verbal communication and is a useful way of analysing people’s experiences or reflections [17]. The interviews were transcribed verbatim and read through several times to gain a comprehensive overview of the content. The next reading focused on identifying passages in the text dealing with experiences of health as well as ill health during their life, with a special focus on occurrences in domestic life and in working life. These parts of the text were divided into meaning units, i.e. condensed sentences in which the original content is preserved. In the coding procedures comparison by gender was made to find similarities and differences. Thereafter, the codes with attached meaning units were sorted into content areas: experiences of health and ill health in relation to domestic life and working life. Finally, codes with similar meanings were grouped into themes [18].

### Ethics

The study was approved by the Regional Ethical Review Board, Umeå University (Dnr 2012–69-31 M) and the Swedish Ethical Review Authority 2020–01950. Prior to the interview, the informants were informed about how the data would be analysed. Thereafter, they gave their consent. The informants were reassured that participation was voluntary and that they could withdraw from the study at any time. The informants were guaranteed confidentiality, and this was secured by disguising their identity in the transcription of the interviews and by replacing their names with a letter and a number after quotations.

## Findings

### Gendered experiences of health

Social determinants of health were identified from how the informants narrated experiences of domestic and working life in relation to their health status. Feelings of well-being, pride, being respected, considered knowledgeable and feeling social worthiness were interpreted as determinants of good health. These determinants were found in the narratives from both women and men and were related to both waged and domestic work, although in somewhat different ways. Health-related experiences formed the following themes: three themes related to working life – “Becoming someone”, ““Being appreciated at work”, “Having control over work” – and one theme – “Having good family relationships” – related to domestic work.

**Fig. 1:**
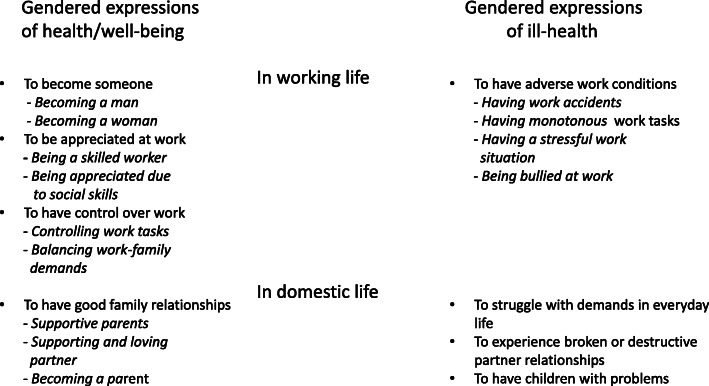
The figure shows the themes and sub-themes in relation to the content areas Experiences of health/well-being and Experiences of ill-health/poor well-being as well as the relation to working and domestic life. Themes are written in plain text and sub-themes in italics

A summary of themes and subthemes is given in Fig. 1.

#### Becoming someone

The theme of “Becoming someone” embraced the informant’s life from leaving school and included the informant’s narratives of occasions/events which were accompanied by a sense of well-being and pride as well as feelings of social worth. Women’s and men’s descriptions of the importance of work showed both similarities and differences, partly depending on which time in life they referred to.

The most important factor for well-being, after leaving school, was to find a job. This was emphasised by both women and men, as a job made them feel grown-up and independent of parents. A young woman who grew up in the countryside and had to move to the town to get a job declared:

“When I left school and got a job I felt very proud. I lost weight after having been overweight during the whole school period” (Woman number 1).

A less fortunate young man with a period of unemployment after leaving school described the importance of a job for well-being like this:

“If you don’t have a job, it feels like you are a burden on society, when you can’t prove yourself, it is like that” (Man number 1).

When growing older and having a family, gendered experiences became more apparent. Gendered expressions are described in the sub-themes “*Becoming a man*” and “*Becoming a woman*”.

For men having a job was still extremely important for their experience of *becoming a man.* The same man who had experienced unemployment when leaving school and then felt like a burden on society expressed how having a high-salary job as an adult boosted his self-confidence:

“It means that you are someone. When you can manage by yourself, it strengthens your self-confidence. I work, I earn money, I can manage by myself” (Man number 1).

To be active with work-tasks was associated with feelings of pride and self-esteem, as described by another man:

“To work is to achieve something, you feel good and simply exist.” (Man number 2).

The importance of work for the men’s self-esteem was attributed to the importance given to work and physically hard work, particular in their families during their upbringing. A man who had a complicated relationship with his father said:

“Work was the only thing that mattered for him. I was very talented at football, but that meant nothing to him” (Man number 3).

Another man said that there had been an ongoing competition between his father, his brothers and himself to outdo each other as to which of them could work the hardest. He had passed on this ideal to his own sons as he said:

“I use my boys as tools, when I can’t do it myself … They are never without anything in their hands. I send them here and there and they accept. They ask, ‘Daddy, do you have anything for us?’ I give them three or four hundred for changing tires on a car or to putting something together” (Man number 2).

#### Becoming a woman

Women’s endeavour to become someone showed similarities to men’s in that having a job was important for their independence but being a “worker” did not seem as closely associated with their self-esteem as it did for men. Instead, social relationships at the workplace seemed to be most important for women’s health. One woman emphasised the spirit in the work team as something important for her well-being at the workplace and how a joyful work situation induced feelings of well-being outside work as well.

“That was the best workplace I ever had. Very good workmates, who supported me when it was worst during our separation. We were such an extraordinary work team. I can enjoy the feeling even today and that’s what all of us who were there then say.” (Woman number 1)

Another woman working in health care said that she liked her job despite the stress and heavy lifting because it gave her the opportunity to help others.

“I like people, I am social. I feel good from helping others and see that you do something helpful for others.” (Woman number 2)

After becoming a mother, the women experienced the need for a health-promoting job to balance against care for the children. Woman number 3 said how important her job was for her well-being and how much joy her work at homes for old people (i.e., residence for elderly people, where they live and receive daily care from assistant nurses) gave her and how she arranged activities to make the residents feel good. At the same time, she said that she was lucky to be able to work part-time to be there for her own children.

#### Being appreciated at work

Being appreciated at work seemed to be important for well-being and feelings of social worth. The narratives about appreciation could come from employers, customers or fellow employees. Gendered expressions of appreciation were seen in situations described as inducing feelings of appreciation. Appreciation due to *“Being a skilled worker*” was mentioned by men when talking about employers who had confidence in their skills and supported their career. Appreciation was mentioned as a key factor for well-being at work, as expressed by these two men

“It was this man [boss], He gave me incredible support. He saw that I could manage tasks he did not expect after such a short time. He inspired me very much and I improved my working skills.” (Man number 1)

“They were very disappointed when I was leaving the town [because of divorce] and gave my notice. My boss said that he had plans to give me a seat in the management team.” (Man number 2)

“*Being appreciated due to social skills*” concludes the women’s descriptions of how appreciation at work induced feelings of well-being. None of the women mentioned their boss when talking about being appreciated at work. Instead, they included the whole staff and mentioned being appreciated due to their social qualities – being one of the team. For example, one woman who had started to work at an old people’s home as temporary staff was later offered employment.

“They like me at the job and want me to stay there and are enlarging my working hours.” (Women number 3)

Appreciation from customers and residents was also experienced as counterbalancing a stressful work situation.

#### Having control over work

To have a clear goal for the job and an opportunity to balance work with family demands was perceived as a basis for health. Gendered expressions in this theme appeared and are sorted into the sub-themes “*Controlling work tasks*” and “*Balancing work-family demands”.*

“*Controlling work tasks*”:men emphasised the importance of being trusted and given freedom to make decisions by themselves on how to do their work.

“I feel good. I don’t need anyone watching over me and saying do this and do that. I have complete control over what to do and I also do it. (Man number 1)

Another man, who had been employed in several jobs earlier and later in life became an entrepreneur as he started a real estate business with a friend, emphasised that the freedom to make his own decisions at work induced feelings of satisfaction despite the long working hours (Man number 3).

“If you want to get something out of the company, you must work very hard … but with the situation I have, working with someone with the same values, I feel fortunate. Maybe that is why we thrive and feel healthy and have the motivation to go to work.” (Man number 3)

Experiencing work control through “*Balancing work-family demands*” was only mentioned by women. For example, a hairdresser with a salon of her own took control over her work situation by hiring a co-worker. This gave her the opportunity to reduce her own working hours, after a long time of stress with a divorce and caring for a sick child. The new work situation with a workmate and one day off every week helped her to find joy in her work again.

#### Having good family relationships

Expressions of health related to good family relationships were primarily aspects of basic security and feelings of well-being through being supported and loved. The theme of family relationships embraced relationships to mothers and fathers during upbringing, relationships to partners and children. In this theme gendered experiences were rare as both men and women emphasised the importance of good family relationships, although women talked more about relationships in general.

Informants emphasised the importance of *supportive parents* during their upbringing for well-being and self-confidence later in life. This was exemplified by parents who encouraged them to follow their own interests when choosing an education, and who pushed and supported their studies. The importance of her mother’s support was also expressed by a woman who got pregnant at a young age and her boyfriend’s mother tried to persuade her to have an abortion. However, her own mother supported her desire to keep the child. As an adult she was very grateful for this choice.

Their parents’ way of being role models and giving support was put forward as the reason for the positive attitude that some informants described as an indefinable feeling of luck and happiness and positive feelings about the future. “I feel fortunate in life” (Woman number 1) and “I am a lucky fool” (Woman number 4) were examples of expressions used.

Having a *supportive and loving partner* was described as a basis for well-being by both women and men. Having support at home could balance negative experiences at work. A man who had experienced bullying at work said:

“I can manage because I have such a fantastic wife. It’s the same girl I met 18 years ago” (Man number 4).

A woman who had started a new relationship after a divorce said that it was extremely good for her well-being.

“He [her new partner] is the person who makes me feel my best, he is a fantastic man and … he also gets along very well with my son” (Woman number 4).

Another woman (number 3) mentioned the good relationship to her ex-husband as important for her own and their children’s well-being after the divorce. They had always been able to communicate with each other and could therefore solve problems related to sharing the care of the children between them after the divorce. In addition, he had a high salary and still contributed financially to a high degree to their children’s upbringing.

*Becoming a parent* was expressed as closely related to health and well-being. Having children was experienced as compensating for earlier hardships, and the children’s well-being was a prerequisite for their own well-being. Negative feelings were put aside and replaced with responsibility for the children’s future, as expressed by one woman: “The children always come first.” She did not regard the work she did at home as being negative for her own health.

“Having four minor children entails hard work, but I have never felt ill from that.” (Woman number 3)

One woman also included a good relationship with her partner’s children from an earlier relationship in her source of well-being.

“We did this trip abroad on my fiftieth birthday, me and my children, my new partner and his children. We were 14 people altogether and I was so happy. It was so nice.” (Woman number 4)

Pride in their children’s achievements was also brought up as inducing well-being.

A man who had not done well at school himself expressed extreme pride in his daughter’s school results. “When she came back to her school class, she helped the teacher during English lessons” (Man number 1). She had lived with him abroad and attended an English-speaking school for a year.

#### Gendered experiences of ill-health

Factors interpreted as causing ill-health were described as feelings of stress, being worn-out, experiencing aches and pain, being disappointed or even ashamed. Gendered expressions of ill-health related to work formed one theme, “Having adverse work conditions”, with the sub-themes *having work accidents, having monotonous work tasks, having a stressful work situation* and *being bullied at work*. The gendered experiences could mostly be attributed to the different sectors in which men and women worked. The men had jobs in industry and technology, while the women worked in the health care and service sectors. Gendered expressions of ill-health related to domestic work formed three themes: “Struggling with everyday demands”, “Experiencing destructive or broken partner relationships”, and “Having children with problems”.

#### Having adverse work conditions

Ill health due to *work accidents* was mentioned only by men. One man had fallen down from a ladder while inspecting a building and incurred multiple fractures and concussion (Man number 3). He had a three-month rehabilitation period and recovered almost completely, which made him feel that he was a lucky man, although he had to do regular exercises to counteract stiffness and pain. Another man (Man number 2) working in physical heavy jobs in industry had been involved in several work accidents and suffered from pain in shoulders and low back, which restricted his working capacity now in his fifties. Despite the chronic pain he claimed, not without pride, that he had never spared himself.

*Having monotonous work tasks* was mentioned as the reason for neck/shoulder pain by a man who had worked soldering circuit cards. As the pain increased despite treatment at the occupational health clinic, he quit that job and slowly recovered. A man who had worked in a business changing car tyres quit his job as he felt that the monotonous work gave him more and more pain (Man number 1).

Ill health due to *having a stressful work situation* was described by both women and men. A man attributed his stress to a job without structure.

“They had a service agreement which guarantees customers help within two to three hours. This meant that I had 20–25 phone calls with customers complaining, because we didn’t have enough staff and I was in charge of the work.” (Man number 3)

Another stressful work situation was described by a woman in health care. She experienced a situation with too much work and too little time to do it, and that work led to exhaustion, both physically and mentally. She often left her job with feelings of guilt about not being able to give the old people the care she wanted. She suffered from recurrent episodes of back pain due to heavy lifting and stress. The working schedules with work every other weekend were also difficult to combine with being a single mother, which induced a lot of stress.

A man told about a long period of *being bullied at work*, when both his boss and some workmates made his life very difficult. The bullying started after a motorcycle accident in which he suffered head injuries. The injuries affected his ability to work fast, and he needed more time than before to manage his tasks. When he returned to his workplace his boss and some of the workmates (all men) treated him as if he was mentally retarded and incompetent. In the end he was not allowed to do anything although the customers were satisfied with his work. He became very depressed and was fired after a time. Being fired was a hard blow to him as he had loved his work and had a strong work ethic.

“When I returned after the accident, my boss instructed all my co-workers that I was not allowed to work in the factory or with tools. When I started working as usual, he called me up to his room and scolded me for a couple of hours, told I shouldn’t think I was special. He just poured abuse on me.” (Man number 4)

#### Struggling with demands in everyday life

The theme of “Struggling with demands in everyday life” is strictly gendered as only women expressed ill-health – extreme tiredness, feelings of disappointment and stress – due to a sense of overload of domestic work. All the women with children and a job mentioned tiredness and unfair sharing of household chores. They perceived that they carried the greatest burden of the housework, and the most common way to deal with this was to reduce their working hours outside the home. A woman with a small business and three children said about the unequal share of housework between her and her husband.

“Well, he helps, but nothing works automatically. You could say that I steer the ship and he starts rowing when I tell him” (Woman number 4).

One woman voiced the frustration that others also experienced from this situation:

*“*Everyone takes me for granted, I have to be everywhere. I can’t go to the toilet and shut the door, I can’t be sick. I feel as if I’ve been taken hostage.” (Woman number 2)

Self-blaming for the unequal share of housework was common and “I have difficulties saying no” (Women number 1 and 2) was generally used as an explanation.

One woman reflected in retrospect on how her situation became like this and understood that it originated from her own childhood.

*“*My mother was a housewife, and the house was always well cleaned. She always baked bread and made jam and so on and she became my role model for how a mother and a family ought to be. Later, I brought that with me to my own family. In the beginning I also liked to do all that” (Woman number 4).

Another woman remembered that she became very disappointed when her mother started to work part-time and was out of the home when she came home from school.

“The week my mother worked it was not as much fun to come home from school because there were no snacks on the table.” (Woman number 1)

Women’s responsibility for relational work extended beyond the own family. Care for a lonely mother or father and for a sick neighbour were also mentioned as tasks to take on. They could not look away from other people’s needs.

None of the men talked about physical or psychological strain due to housework. Instead, one man told about his experiences of support from his wife when he had a hard time at work. Another man claimed that he became stressed from staying at home with the children.

“When I am at home taking care of the children, I feel OK, it’s quite a lot of work, but I also become restless and think that I must have something else to do than take care of the children.” (Man number 1)

#### Experiencing broken or destructive partner relationships

If good relationships were expressed in relation to positive health, problematic and broken relationships were expressed as detrimental to health. This was apparent in interviews with both women and men. Negative feelings associated with broken relationships varied from profound disappointment and loss of self-confidence to depression and shame.

A strained relationship over a longer or shorter period mostly preceded a separation or divorce, and the women seemed to put more effort into trying to save the marriage. They also cited the children’s best interests as the reason for the struggle.

“I held out for seven years, until my son was twelve. You know, you try for the family’s sake. But in the end, you have to think about yourself also and learn to say no.” (Woman number 1).

A woman described how her relationship with a very jealous man almost destroyed her self-esteem. “I felt that I was always in the dock as the accused, and he was the prosecutor …. It felt as if you were an apple, he took a chew every time and, in the end, there was only the core left” (Woman number 4). Another woman expressed both fear and tiredness due to her difficulties in breaking up from a destructive relationship with a depressed man.

“It would not help if I moved to another address in this town, he will just look me up. I think I have to move to another town to really break up with him. But at the same time, I don’t know … then I will become so lonely.” (Woman number 5)

Disappointment at being betrayed and left by a partner was described in parallel with feelings of social failure and a ruined economy.

“The broken relationships are what caused me the largest dips. The last one was a great, great disappointment and caused this plunge.” (Man number 2)

This man had large debts after his divorce, which further contributed to his depression.

#### Having children with problems

Having a child with ill-health was a topic emphasised as having negative effects on the parents’ own health. A constant worry parallel to the extended care that a sick child required increased the already high workload and could result in extreme fatigue and depressive feelings. For one woman the situation contributed to the divorce, and she described her tiredness during this period as “having a social puncture” (Woman number 4). She had no energy left to be socially active.

“My youngest son suffered from a depression three years ago. It ended in a crash for all of us … .When it comes to your children, and as I understand it grandchildren as well, you fight until you go down and a little bit more.” (Woman number 4)

Similarly, parents, who had a child with drug abuse and a child subjected to sexual abuse also told us about the strain this situation put on the whole family and everybody’s well-being.

## Discussion

A major finding in our analysis was the importance of gender relations in society for the participants’ experiences of work-related determinants of health during life. The gender order in society defines the gendered structure of the labour market, for example, in which men and women to a large extent are segregated into different occupations [4, 5]. First, however, our findings about determinants of positive health will be discussed.

A major theme in our interviews was the health-promoting experiences related to being employed. Already in young age, finding a paid job after leaving school, rather than being unemployed, was held up as being important for health. Both the men and the women felt that they “became someone” through the waged work.

The interviews here are in line with our consistent findings in NoSCo, indicating the negative health consequences for both men and women of unemployment from young age onwards [15]. One of the explanations for the negative health consequences of unemployment was a model defined by Marie Jahoda in her studies of unemployed communities in Austria during the early 1930s [19]. She identified that a paid job had, besides financial importance, several latent functions including giving the day a time structure, providing opportunities for social contact with other people, contributing to status and personal identity for the individual, and providing an opportunity to strive towards collective purposes and shared experience. Unemployment, when these latent functions are lacking, can thus result in ill-health. Some of these latent functions connected to paid work were expressed in a qualitative study of elderly women in Sweden [11]. The study showed the positive impact of work for health, as paid work gave a meaning to the women, a room of their own as well as self-esteem and social support. In addition, earning money gave them independence. For both men and women, it was of major importance for health to be appreciated at work. But there were gendered patterns, as for men it was important to be appreciated for one’s skills while for women good social relations were of crucial importance. These gendered experiences could be related to the gender regime of the Swedish society, dividing men and women into different occupations and different responsibilities for domestic work [4, 5]. Relational femininity (constructed from responsibility taking, caring about others, striving for good social relations) is of major importance in women-dominated occupations which require social skills and relational work (health care, elderly care, school, nursing homes etc) [4, 5]. Relational-oriented femininity is constructed from traditional ideals about a desirable femininity in society, based on social relations at work (exemplified in the subthemes *Being appreciated due to social skills* and *Balancing work-family demands*) as well as in domestic life (exemplified by the theme “Struggling with demands in everyday life”). The material basis for the construction of this femininity is a society dominated by hegemonic masculinity, in which femininity is devaluated and women are supposed to perform the low paid relational work as well as the unpaid domestic work [4, 5].

Thus, even in a gender-equal country like Sweden, it is still most often women who have the main responsibility for domestic work [20]. Having children with problems added to the extra burden of domestic work as expressed by women in our study. A strategy for women to achieve a balance between waged and domestic work is to work part-time (23% as compared to 9% among Swedish men), resulting in a poorer financial position and a smaller pension [20]. Gender inequality in the couple relationship was also documented in the questionnaires at age 30 and 43 in the follow-up of NoSCo. Women overall had greater responsibility for domestic work, and gender inequalities in the domestic sphere were shown to be related to mental ill health among both men and women [12]. Another qualitative study from NoSCo (based on another sample of participants) at age 47 also showed that unequal gender relations were an important part of how the domestic work was organised and related to experiences of mental illness among women and men. Among women the high burden of housework was experienced as an obstacle to experiencing good health. Among men the experience of being trapped in an outmoded masculinity was related to feelings of stress [12].

Construction of a dominating and violent masculinity was visible in quotations from women in our study. The theme “Experiencing broken or destructive partner relationship” illustrates the negative health consequences of men’s violence against women. In this study, the exposed women mostly talked about psychological violence, but violence could also be physical and/or sexual. Men’s violence against women is a global cause of serious somatic, mental, sexual, and reproductive health problems for women [21].

Our findings about work-related ill health illustrate the importance of the gendered physical work environment and the increased risk of accidents in men-dominated jobs. A hard-working masculinity could be identified, striving to fulfil the masculine ideals of working-class men by working hard and accepting all available jobs, despite hazardous work environments leading to severe work-related accidents. Hard-working masculinity was further expressed as never spare oneself, despite hard physical work and workplace accidents leading to chronic pain (theme “Adverse working conditions”). Hard-working masculinity may also lead to broken relationships (in the theme “Experiencing broken or destructive relationships”) as the man could be experienced as absent in family life as well as in domestic work [12].

Agency was expressed as quitting a job, due to monotonous work tasks which led to neck/shoulder pain. Our subtheme: *Becoming a man* identified how waged work was a prerequisite for self-confidence and identity among men in the study. The social history of reproducing this masculinity was illustrated by a father who competed with his sons about who worked hardest and the son in turn replicated the same ideals to his sons. Hard-working masculinity also included being a skilled worker as expressed in the theme “Being appreciated at work”. A skilled worker could be trusted and given freedom to make his own decisions, as expressed by men in the theme “Having control over work”.

A report from the Swedish Work Environment Authority [22] also illustrates that masculine norms and attitudes towards safety and risk-taking could be one explanation for the increased risk of severe work-related accidents among men, especially in industrial workplaces. Masculine ideals among these workers consist of physical strength, endurance, ability to sustain pain and practical skills, which could also be an explanation why the men in our study emphasised the need to be appreciated due to their skills.

Gendered dimensions are also related to some of the other adverse work environments in our study (even though there were no gendered experiences of them among our participants). Stress (often operationalised as job strain) is most common in women-dominated workplaces [23] and work tasks that are repetitive and monotonous are more common among women [24]. One man in our study reported long-standing negative health consequences of being exposed to workplace bullying. A longitudinal Norwegian study found that workplace bullying is a serious long-term threat to the health of men and called for more gender research in the field [25].

Admitting being bullied may be regarded as breaking the hegemonic norm of being a strong and non-vulnerable man. Other examples of a possible norm breaking masculinity or femininity could be when similar experiences were expressed by men and women. For example, both men and women in our study experienced control at work as positive for health. Similar conclusions were drawn from a quantitative meta-analysis [23] which shows that there is substantial empirical evidence that employees, both men and women, who report lack of decision latitude and job strain will experience increasing depressive symptoms over time. Gender similarities were also identified in our study in relation to the importance of having good family relationships, even though the women talked more often about relationships in general. Our findings are in line with a meta-analysis, which evaluated the relation between social support and depression in youth and found that gender differences were largely absent [26].

### Methodological considerations

The participants in this study were recruited from a population-based cohort study, which had been followed with questionnaires and interviews over a long period. The nine participants in this interview study were strategically chosen to get the best possible variations in experiences. The longitudinal design is considered valuable for the credibility of the findings as the participants have shown an interest in the study through their participation over the years and trust the principal investigator of the cohort study (AH). Credibility of findings also presupposes confidence in how well interviews and analyses address the intended issue. The participants in our study were asked to reflect on their lives and narrate specific events in relation to perceived health. The interviews were long and rich in content and concerned both working and domestic life. To increase the trustworthiness of the study and to address the researchers’ preunderstanding, the interpretations and formulations of themes were discussed and negotiated among the authors during the analysis process. The authors also discussed the findings in seminars with researchers in other research fields.

Examples of participants’ voices are given in expressive quotations, in which all participants were represented.

## Conclusions

Gendered determinants of health and ill-health were identified in both working and domestic life during life. Public health policy needs to challenge the gender order in society, which defines the gendered structure of the labour market as well as the gendered relations in domestic life.

## Data Availability

Data are not freely available. The Swedish Data Protection Act (1998:204) does not permit sensitive data on humans (as in our interviews) to be freely shared. After ethical approval the anonymous data set could be obtained on request to Umeå University after their confidentiality examination.
